# Interprofessional education or silo education?

**DOI:** 10.36834/cmej.v8i4.41987

**Published:** 2020-08-06

**Authors:** Hugh James Brocklebank, Tanisha Jowsey

**Affiliations:** 1 University of Auckland, New Zealand

**Figure:**
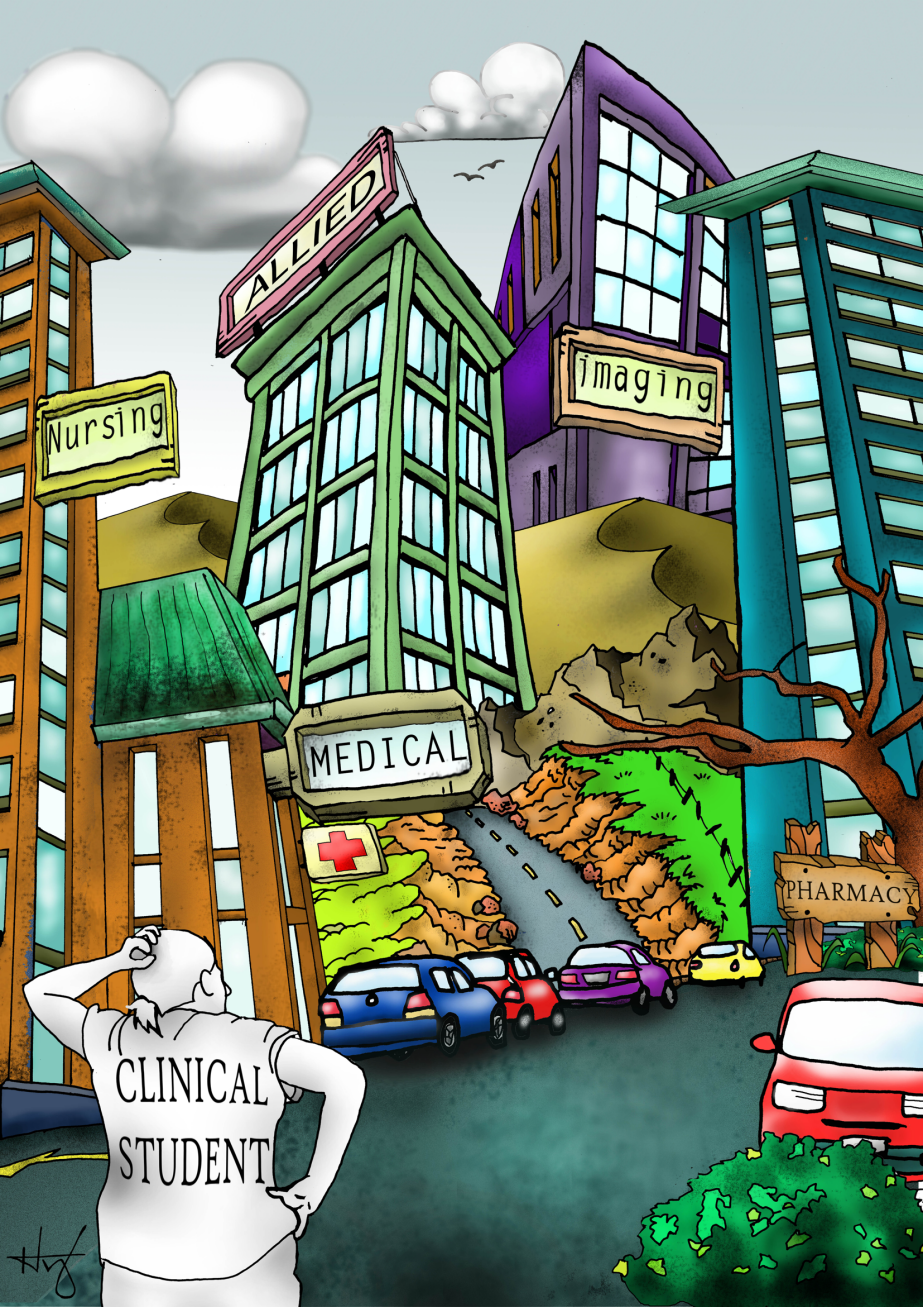


This image depicts the difficulties that practitioners have in working in interprofessional teams when they train in separate buildings and don't learn together. It was hand drawn and coloured using Adobe Photoshop 2015.

